# Assessment of Immunotoxicity of Dextran Coated Ferrite Nanoparticles in Albino Mice

**DOI:** 10.1155/2015/518527

**Published:** 2015-10-20

**Authors:** Santhakumar Syama, Viswanathan Gayathri, Parayanthala Valappil Mohanan

**Affiliations:** Toxicology Division, Biomedical Technology Wing, Sree Chitra Tirunal Institute for Medical Sciences and Technology, Thiruvananthapuram, Kerala 695 012, India

## Abstract

In this study, dextran coated ferrite nanoparticles (DFNPs) of size <25 nm were synthesized, characterized, and evaluated for cytotoxicity, immunotoxicity, and oxidative stress by *in vitro* and *in vivo* methods. Cytotoxicity was performed *in vitro* using splenocytes with different concentrations of DFNPs. Gene expression of selected cytokines (IL-1, IL-10, and TNF *β*) secretion by splenocytes was evaluated. Also, 100 mg of DFNPs was injected intraperitoneally to 18 albino mice for immunological stimulations. Six animals each were sacrificed at the end of 7, 14, and 21 days. Spleen was subjected to immunotoxic response and liver was analyzed for antioxidant parameters (lipid peroxidation, reduced glutathione, glutathione peroxidase, superoxide dismutase, and glutathione reductase). The results indicated that DFNPs failed to induce any immunological reactions and no significant alternation in antioxidant defense mechanism. Also, mRNA expression of the cytokines revealed an increase in IL-10 expression and subsequent decreased expression of IL-1 and TNF *β*. Eventually, DNA sequencing of liver actin gene revealed base alteration in nonconserved regions (10–20 bases) of all the treated groups when compared to control samples. Hence, it can be concluded that the DFNPs were nontoxic at the cellular level and nonimmunotoxic when exposed intraperitoneally to mice.

## 1. Introduction

Nanoparticles are diverse class of small-scale (<100 nm) substances with novel properties like small size, large surface area, specific shape, and surface activity [1]. Toxicity of nanomaterials refers to the interaction of nanomaterials with the biological systems and induction of toxic responses. Once inside the body, they get distributed to various organs or may remain in the same site and can be structurally modified or metabolized. When inhaled, they can translocate out of the respiratory tract via different pathways and mechanisms. When ingested, systemic uptake of nanomaterials via lymph can occur. When in blood circulation, they can be distributed throughout the organism and will be taken up by liver, spleen, bone marrow, heart, and other major organs [2, 3]. Depending on the duration of exposure, these materials can translocate from circulation to internal organs. Hence, the potential for significant biological response at each of these sites requires investigation [4].

Magnetic nanoparticles are widely used in biomedical applications such as contrast agents in magnetic resonance imaging (MRI) [1] and in tissue repairing [5], detoxification of biological fluids, hyperthermia [6], drug delivery [7], cell separation [8], drug targeting [2], and so forth. Ferrite particles coated with biocompatible phases like hydroxyapatite are employed for hyperthermia treatment of cancer [9]. As bare metal and metal oxide nanoparticles are toxic to biological systems, coating with biocompatible phases is often done to increase the biomimetic nature of the particles suiting for various biomedical applications. Dextran (C_6_H_10_O_5_), a branched polysaccharide, obtained by microbiological synthesis is used to coat iron nanoparticles. Dextran being nontoxic, biodegradable, and hydrophilic may facilitate the intracellular uptake of dextran coated magnetic iron particles [3, 4].

The major toxicological issue associated with the manufactured nanomaterials is that some of them are redox active; they can transport across cell membranes and interact with subcellular organelles. As a consequence of all these properties, nanoparticles can have direct interaction with individual target cells, either with the external membrane or inside the cell at the site of action. Advances in nanotechnology led to the exposure of humans to engineered nanomaterials and hence it became necessary to evaluate the potential human health effects before these materials are fully exploited [1, 2].

Experimental evidences have shown that metal and metal oxide nanoparticles induced DNA damage and apoptosis through ROS generation and oxidative stress [10, 11]. It has been reported that magnetic nanoparticles administered intraperitoneally can cross blood-brain barrier where they have the potential to affect cerebral functions [12]. Due to the high reactivity of ROS, most cellular components are likely to be the targets of oxidative damage: lipid peroxidation, protein oxidation, GSH depletion, and DNA single strand breaks. All of these events ultimately lead to cellular dysfunction and injury [13]. For this reason, antioxidant enzymes are vital markers for oxidative stress induced in the body. Aerobic organisms possess antioxidant defense systems that deal with the removal of ROS. As long as there exists a balance between oxidative stress and antioxidant defense system, human body is maintained in an optimal health state.

Antioxidant defense system includes both low-molecular-weight free radical scavengers, such as the tripeptide glutathione (GSH), as well as antioxidant enzymes such as superoxide dismutase (SOD), glutathione reductase (GR), and glutathione peroxidase (GPx) [3].

The immunotoxicology of engineered nanoparticles is a growing concern in the present era. The potential immunotoxicity and mechanisms of action of these particles have not received sufficient attention. Nanoparticles can interact with immune system in several ways and can enhance or suppress immune function depending on their physicochemical characteristics [14]. It was reported that the surface modification of carbon nanotubes recognizes scavenger receptor and alleviates NF-*κ*B activation and reduces its immunotoxicity [15]. Similarly, shell-cross-linked knedel-like nanoparticles induced lower immunotoxicity than their non-cross-linked analogs as was demonstrated by Elsabahy et al. [16].

Here, DFNPs intended to be used for targeted drug delivery applications were in-house synthesized and characterized. The primary focus of the present study was to examine the immunotoxic potential and cellular response of DFNPs.

## 2. Materials and Methods

### 2.1. Chemicals

Thiobarbituric acid (TBA), reduced glutathione (GSH), oxidized glutathione (GSSG), and dithiobis-2-nitrobenzoic acid (DTNB) were purchased from Sigma Chemical Co., St. Louis, MO, USA. Pyrogallol (PG), diethylene triamine pentaacetic acid (DTPA), and trichloroacetic acid (TCA) were purchased from Merck, Germany. RNase, ethanol, bromophenol blue, ethidium bromide, 100 bp DNA ladder, and Taq polymerase were purchased from Fermentas, USA; mouse oligonucleotide primers for interleukin 1 (IL 1), interleukin 10 (IL 10), tumour necrosis factor beta (TNF *β*), and beta actin (*β* actin) were procured from Eurogentec, Belgium; RPMI-1640 from Himedia, Mumbai, India; RT^2^ SYBR green ROX q PCR master mix, RNeasy lipid tissue mini kit, Qiazol lysis reagent, and RT^2^ first strands kit were from Qiagen, Hilden, Germany, GenElute mammalian genomic DNA Miniprep kit was from Sigma/Aldrich, USA. Mouse T and B cell selection kit was from Stem Cell Technologies Inc., Canada. ^3^H-thymidine was from BRIT, India. All the other chemicals used were of analytical grade and were purchased from qualified local vendors.

### 2.2. Equipment

The equipment used was as follows: spectrophotometer (Shimadzu, Japan), laminar air flow (Mark Air particulars, India), incubator shaker (New Brunswick Scientific, USA), biophotometer (Eppendorf, Germany), and steam sterilizer (Nat Steel, India).

### 2.3. Animal Husbandry and Welfare

All animals were handled humanely, without making pain or distress and with due care for their welfare. The care and management of the animals will comply with the regulations of the Committee for the Purpose of Control and Supervision of Experimental Animals (CPCSEA), Government of India. All the animal experiments were carried out after prior approval from Institutional Animal Ethics Committee and in accordance with approved institutional protocol.

Healthy albino mice were used for the study. The body weight ranges between 17 and 23 g and was maintained in a 12 h light/dark cycle at a constant temperature of 22 ± 3°C with free access to standard pellet diet and water. Individual animals were identified with picric acid marks on mice. In addition to this, each animal cage was identified by labels having details such as experiment number, name, animal number(s), and date of experiment. All the animals were acclimatized for a period of 5 days before initiation of experiment.

### 2.4. Synthesis of Dextran Coated Ferrite Nanoparticles (DFNPs)

DFNPs were prepared using the coprecipitation method. Briefly, the stoichiometric mixtures of FeCl_3_ and FeCl_2_·4H_2_O (Fe^3+^/Fe^2+^ : 2 : 1) were heated at 70°C in N_2_ atmosphere. Ferrite nanoparticles were precipitated by the addition of 3 M NaOH dropwise for 1 h followed by heating with stirring for about another 1 hr. The precipitate was then washed with deionized water three times to get uniformly dispersed spherical magnetite particles. All reactions were carried out in N_2_ atmosphere to prevent oxidation of magnetite to magnemite. Surface coating of ferrite nanoparticles with dextran was done by overnight stirring (at 37°C) of ferrite nanoparticles in a solution of dextran of appropriate concentration. The precipitate was then washed and lyophilized to get dextran coated ferrite nanoparticles (DFNPs). The size determination of synthesized DFNPs was studied by Transmission Electron Microscopy (TEM) analysis. Further characterization of DFNPs was reported by us [17–19].

### 2.5. Cytotoxicity

The cytotoxicity assay was performed by MTT assay using spleen cells [20]. The spleen cells were seeded at a density of 20,000 cells/well in a 96-well plate at 37°C in 5% CO_2_ atmosphere. After 24 h of culture, 200, 400, 600, 800, and 1000 *μ*g/mL of DFNPs (in triplicate, cells alone as negative control) were added onto the suspension culture of spleen cells. The cells were incubated at 37 ± 1°C for 24 ± 1 h and examined microscopically for morphological changes and quantitated by MTT assay. 20 *μ*L of MTT dye solution (5 mg/mL in phosphate buffer pH 7.4) was added to each well. After 4 h of incubation, the MTT was removed and formazan crystals formed were solubilized with 200 *μ*L of DMSO. The absorbance of each well was read on a microplate reader (ELx 808iu ultramicroplate reader, Bio-Tek instruments, USA) at 540 nm. The relative cell viability (%) was calculated.

### 2.6. Reactive Oxygen Species (ROS)

The generation of ROS was monitored by employing 2,7,dichlorodihydrofluorescein diacetate (H_2_DCFDA) [Invitrogen] which is nonfluorescent unless oxidized by the intracellular ROS. A dose dependent measurement of the ROS generation was done by preincubating 20,000–40,000 spleen cells with H_2_DCFDA at a concentration of 100 *μ*M. Then, the cells were exposed to 200, 400, 600, 800, and 1000 *μ*g/mL of DFNPs (in triplicate) for 2 h, at 37°C. Cells were then washed in serum-free medium and resulting fluorescence intensity was read in a fluorescence microplate reader using an excitation wavelength of 488 nm and emission wavelength of 535 nm. The values were normalized to the negative control (spleen cells alone).

### 2.7. Immunotoxicity

Immunotoxicity is to assess the potential adverse effects on the immune system, which is an important component of the overall evaluation of drug/chemical/nanomaterial toxicity. In this study, 100 mg of DFNPs was injected intraperitoneally to 18 albino mice for immunological stimulations. Six animals each were sacrificed at 0 days (animals not exposed to nanoparticles) and at the end of 7, 14, and 21 days [total 24 albino mice] after exposure. At the end of each observation period, gross necropsy was carried out, and spleen and liver were collected for the following studies.

### 2.8. T and B Lymphocyte Proliferation Assay

Spleen was excised from experimental animals and splenocytes were isolated to study the cell proliferation by tritiated (^3^H) thymidine incorporation assay. Viability of splenocytes was assessed using trypan blue dye exclusion method. Single cell splenocytes suspension was used to separate T and B lymphocytes using kit (EasySep B cell enrichment kit, T cell enrichment kit) according to the protocol described in the kit using an automated cell separator (ROBOSEP). Both the T and B lymphocytes (2,00,000 cells/well) were cultured in a 96-well plate in RPMI-1640 medium supplemented with 10% FBS, streptomycin (100 *μ*g/mL), and penicillin (100 units/mL) for 24 h at 37°C in a CO_2_ incubator. After 48 h of incubation, ^3^H-thymidine at a concentration of 1 *μ*Ci/mL was added to each well and incubated further for 24 h at 37°C. Cells were harvested after 72 h and radioactivity in terms of counts per minute (cpm) was measured by liquid scintillation counter [21, 22].

### 2.9. Preparation of Liver Homogenate

Liver was washed in normal saline and immediately placed in ice bath and homogenized. 10% liver tissue homogenate was prepared in phosphate buffer (0.1 M, pH 7.4) using an ice-chilled glass homogenizing vessel in a rotor stator homogenizer at 900 rpm. This was centrifuged at 3500 rpm for 10 min at 4°C. The resultant supernatants were maintained in an ice bath until being used for the estimation of total protein, lipid peroxidation, reduced glutathione, and antioxidant enzymes (glutathione reductase, glutathione peroxidase, and superoxide dismutase) using standard protocols with slight modifications.

### 2.10. Total Protein

Total proteins in the liver homogenate of mice exposed to DFNPs were estimated by the method of [23], using bovine serum albumin as standard.

### 2.11. Lipid Peroxidation

The extent of lipid peroxidation (LPO) in the liver homogenate of mice exposed to DFNPs was determined as the concentration of malondialdehyde (MDA) generated by the thiobarbituric acid reactive substances (TBARS), as described by [24]. The amount of MDA formed was measured spectrophotometrically at 532 nm.

### 2.12. Reduced Glutathione

Reduced glutathione (GSH) level in the liver homogenate of mice exposed to DFNPs was determined by the method of [25], with slight modifications in which Ellman's reagent or DTNB (5,5′-dithiobis-(2-nitrobenzoic acid)) reacts with GSH to form a spectrophotometrically detectable product at 412 nm. The change in absorbance at 412 nm is a linear function of the GSH concentration in the reaction mixture and is based on the reaction of GSH with DTNB to give a compound 5-thionitrobenzoic acid (TNB) that is absorbed at 412 nm. The amount of GSH was expressed as nmol/mg protein.

### 2.13. Antioxidant Enzymes

The glutathione reductase (GR) activity in liver homogenate of mice exposed to DFNPs was determined by measuring the reduction of GSSG in the presence of NADPH as described by [26]. Thus, one GR unit is defined as the reduction of one *μ*M of GSSG per minute at 25°C and pH 7.6.

Activity of glutathione peroxidase (GPx) in the liver homogenate of mice exposed to DFNPs was assayed by the method described by [27]. The remaining GSH after the enzyme catalyzed reaction was complexed with DTNB, which absorbs at maximum wavelength of 412 nm. Enzyme activity was expressed as *μ*g of GSH consumed/min/mg protein.

The superoxide dismutase (SOD) in liver homogenate of mice exposed to DFNPs was done using modified pyrogallol autoxidation method spectrophotometrically measured at 420 nm [28].

All measurements were carried out using UV Spectrophotometer-1601, Shimadzu, Japan.

### 2.14. Real Time PCR Analysis for Determining m-RNA of Specific Cytokines

Total m-RNA was isolated from splenocytes exposed to DFNPs (200, 600, and 800 *μ*g/mL) and bare ferrite nanoparticles (600, 800 *μ*g/mL), following the manufacturer's protocol, using Trizol reagent (Sigma, USA). 150 ng of m-RNA was used for c-DNA synthesis of IL-10, IL-1, TNF *β*, and *β* actin in a reaction volume of 20 *μ*L using RT^2^ first strand Kit (Qiagen, Germany) and the synthesis was carried out on the Eppendorf master cycler, Germany. The mouse oligonucleotide forward and reverse primer sequences used to determine specific m-RNA gene expressions are depicted in Table 1. The real time PCR reaction was carried out with RT^2^ SYBR green ROX q PCR master mix of total reaction volume of 25 *μ*L, and real time PCR amplifications were done using a Chromo 4 System, Bio-Rad (MJ Research, CA) for 40 cycles as per manufacturer's protocol. The level of gene expression is reported as the ratio between the mRNA level of the target gene and the *β* actin, a reference gene using the comparative 2^ΔΔCt^ method [29].

### 2.15. Nuclear DNA (nDNA) Isolation and Amplification of *β* Actin

nDNA was isolated from liver tissues of mice used for immunotoxicity studies, as per the manufacturer's protocol (Sigma, USA). The quantity and quality of the isolated nDNA were estimated (nanodrop, Eppendorf, Germany). nDNA isolated from* in vivo* experimental groups was amplified using mouse *β* actin specific forward primer (f-GCGTGGGGACAGCCGCATCTT) and reverse primer (r-ATCGGCAGAAGGGGCGGAGA) (Eurogentec, Belgium, Accession number HQ675031.1) at a concentration of 100 ng/*μ*L per reaction mixture. PCR of nDNA was carried out as per standard conditions [30] in Eppendorf master cycler; Germany. Purity and integrity of the amplified products were checked by purity factor (260/280 nm) and agarose gel electrophoresis.

### 2.16. Sequence Analysis of *β* Actin Nuclear DNA (nDNA)

The amplified PCR product (*β* actin) from the above immunotoxicity studies was purified with a QIAquick PCR purification kit (Qiagen, Hilden, Germany) according to the manufacturer's instructions. DNA sequencing was done by Sanger's dideoxy chain termination method [31]. *β* actin nDNA products were sequenced using mouse *β* actin forward and reverse primers in AB1 prism 3730 Genetic analyzer DNA sequencer with a Big Dye terminator cycle sequencing ready reaction kit (Applied Biosystems Japan Co., Ltd., Tokyo, Japan). ABI Sequence Scanner was used for sequencing and alignment of sequence was done using Bioedit software. After sequence analysis and alignment, using sequence navigator program version 2.1, all sequences were submitted to NCBI website (http://blast.ncbi.nlm.nih.gov/Blast.cgi) and BLAST sequence similarity search was conducted.

### 2.17. Statistical Analysis

All values are expressed as Mean ± SD unless otherwise stated. Statistical significance between the control and experimental values was compared by Student's *t*-test. For all comparisons, *P* < 0.05 was considered significant.

## 3. Results

### 3.1. Synthesis and Characterization of DFNPs

**Figure 1:**
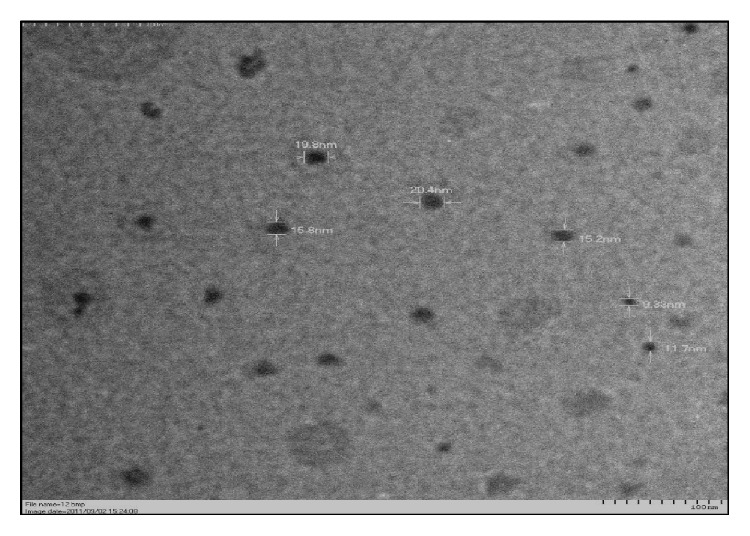
TEM image of DFNPs (X30K).

Ferrite nanoparticles were prepared by the standard coprecipitation method and coated with dextran to yield DFNPs. The detailed characterization of this material was published by [17, 18]. The Transmission Electron Microscopic (TEM) images indicate that a very uniform size distribution of DFNPs particles and was found to be less than 25 nm (Figure 1).

### 3.2. Cytotoxicity

**Figure 2:**
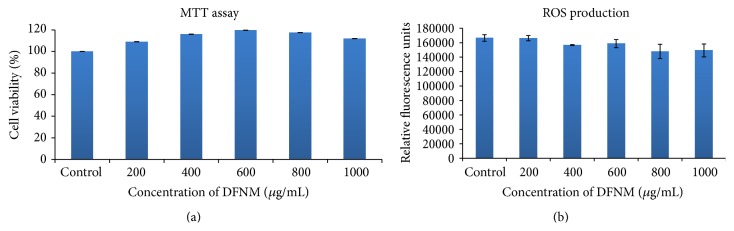
(a) Viability of splenocytes incubated with DFNPs (values are Mean ± SD, *n* = 3). (b) ROS production in splenocytes exposed to DFNPs (values are Mean ± SD, *n* = 3).

Cytotoxicity of DFNPs was evaluated in cultured splenocytes; the results are depicted in Figure 2(a). No marked decrease in cell viability was observed after 24 hrs of exposure for all the concentration tested.

### 3.3. Reactive Oxygen Species

It is well known that most of the manufactured nanoparticles cause cell injury by increasing reactive oxygen species formation. Thus, the measurement of ROS upon exposure to DFNPs is far more important. The amount of ROS generated after 24 hrs of exposure to DFNPs is illustrated in Figure 2(b). The results of the study suggest that the ROS generation in all the samples was well comparable with control values and was not statistically significant.

### 3.4. Immunotoxicity

The animals exposed to DFNPs were monitored throughout the experimental period for any unusual changes. The general physical conditions of the experimental animals were normal. The increase in body weight and feed intake was normal and none of the animals showed any abnormality or behavioral changes during the experimental period. Gross examination of carcasses of control and DFNPs exposed mice did not reveal any abnormality in the organs examined.

### 3.5. T and B Lymphocyte Proliferation Assay

**Figure 3:**
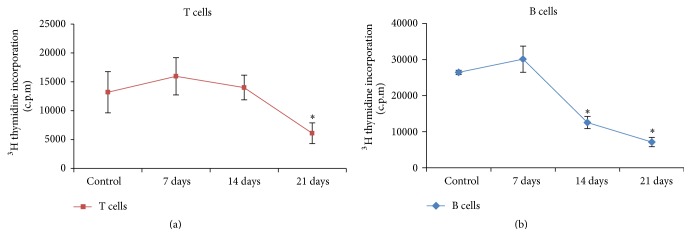
Proliferative activity in spleen cells of exposed to DFNPs. (a) T lymphocyte cells. (b) B lymphocyte cells (values are Mean ± SD, *n* = 5);  ^*∗*^statistically significant *P* < 0.05.

Lymphocyte proliferation is one of the markers to predict the immune response induced upon nanoparticle exposure. The proliferation assay was evaluated in both T and B lymphocytes isolated from spleen of animals exposed to DFNPs for different time periods. The result of the proliferation assay is shown in Figure 3. It was observed that, at 7 days after exposure, a slight increase in thymidine incorporation was observed when compared to control although the effect was statistically insignificant. Decrease in lymphocyte proliferation was observed for T cells after 21 days of exposure and after 14 and 21 days for B cells. The differences were statistically significant on comparison to control.

### 3.6. Lipid Peroxidation

**Figure 4:**
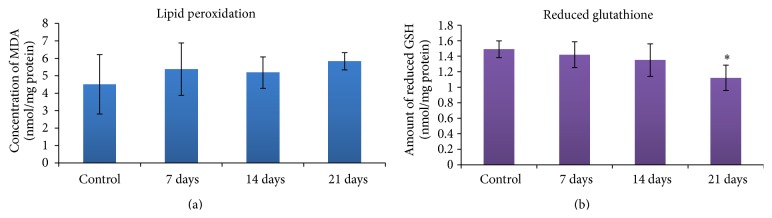
(a) Level of malondialdehyde in liver cells of mice exposed to DFNPs (values are Mean ± SD, *n* = 3). (b) Level of GSH in liver cells of mice exposed to DFNPs (values are Mean ± SD, *n* = 3);  ^*∗*^statistically significant *P* < 0.05.

Figure 4(a) shows the malondialdehyde (MDA) production in the liver homogenate of mice after exposure to DFNPs. The results indicate that the level of LPO production in the control and 21 days postexposure group was 4.51 ± 1.7 and 5.83 ± 0.5 nmoles/mg proteins, respectively. It was observed that the level of LPO was not significantly increased at any time periods (7, 14, and 21 days after exposure) when compared to control.

### 3.7. Reduced Glutathione

The level of reduced glutathione (GSH) in the liver homogenate of mice exposed to DFNPs is illustrated in Figure 4(b). The result suggests that a time dependent decrease in the level of GSH was observed in the liver homogenates of mice with the significant decrease in 21 days postexposure group (1.12 ± 0.2) when compared to control (1.49 ± 0.1).

### 3.8. Antioxidant Enzymes

**Figure 5:**
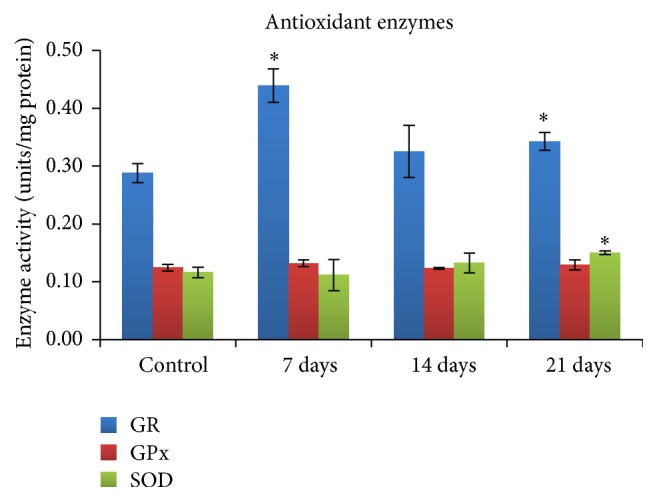
Level of GR, GPx, and SOD in liver cells of mice exposed to DFNPs (values are Mean ± SD, *n* = 3);  ^*∗*^statistically significant *P* < 0.05.

The level of glutathione reductase (GR), glutathione peroxidase (GPx), and superoxide dismutase (SOD) was illustrated in Figure 5. The result of the study indicates that the production of GR was significantly increased at the end of 7 days and decreased at the end of 21 days after exposure of DFNPs (0.34 ± 0.02) in mice when compared to control (0.29 ± 0.02). Similarly, there was significant increase in the production of SOD in the liver homogenate of mice 21 days after exposure of DFNPs (0.15 ± 0.00) when compared to control (0.12 ± 0.01). In addition, there was no increase in the level of GPx observed at any time points which was well comparable to control values.

### 3.9. Real Time PCR Analysis for Determining m-RNA of Specific Cytokines

**Figure 6:**
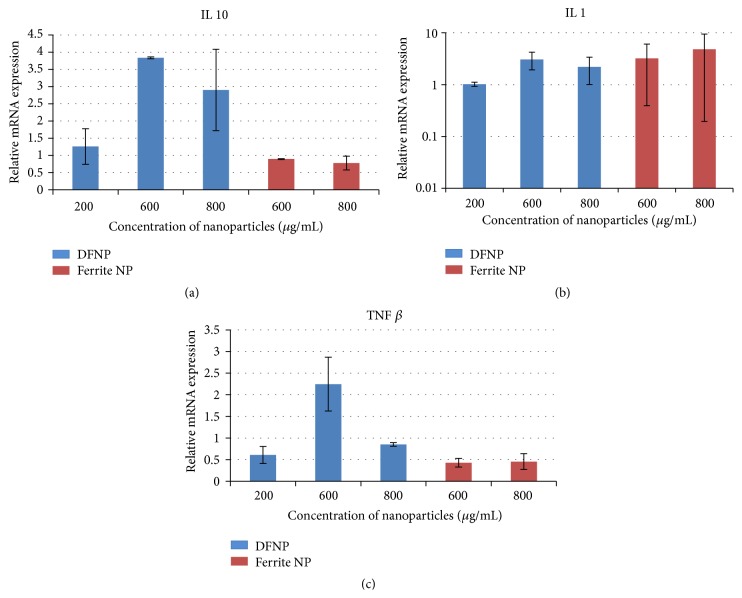
(a) Relative mRNA expression (IL 10) when splenocytes were exposed to different concentrations of DFNPs (values are Mean ± SE, *n* = 3). (b) Relative mRNA expression (IL 1) when splenocytes were exposed to different concentrations of DFNPs (values are Mean ± SE, *n* = 3). (c) Relative mRNA expression (TNF *β*) when splenocytes were exposed to different concentrations of DFNPs (values are Mean ± SE, *n* = 3).

As evident from Figure 6(a), IL-10 mRNA expression was increased by 3-fold in 600 *μ*g/mL DFNPs cells and 2-fold in 800 *μ*g DFNPs exposed cells on comparison of cells treated with similar concentration of bare ferrite particles. No marked difference was noticed in IL-1 mRNA expression for both DFNPs and bare ferrite nanoparticles treated cells (Figure 6(b)). Eventually, there was a dramatic decrease in m-RNA expression of TNF *β* in DFNPs particles in a concentration dependent manner when compared with bare ferrite particles. Differences were observed in both concentrations (600 *μ*g and 800 *μ*g/mL) of DFNPs when compared to bare ferrite nanoparticles (Figure 6(c)). The values are expressed in Mean ± SE.

### 3.10. Sequence Analysis of *β* Actin Nuclear DNA (nDNA)

**Figure 7:**
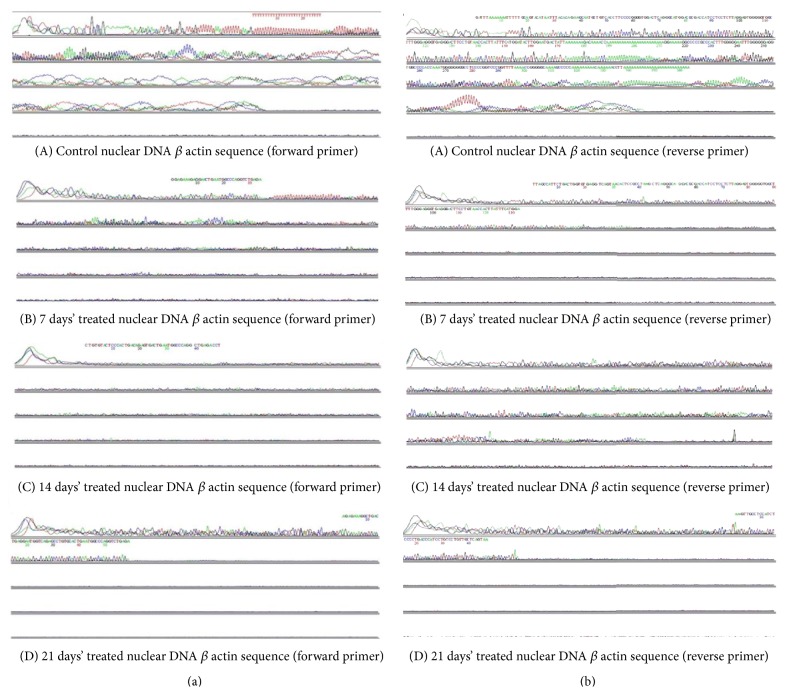
Sequence analysis of cytoskeletal *β* actin from mouse liver.

From Figure 7(a) ((B), (C), and (D)) (treated groups, forward *β* actin primer) when compared to Figure 7(a) (A) (Control group, forward *β* actin primer) it was evident that bases 10 to 20 of the sequence were altered in 7, 14, and 21 days' treated mice liver, respectively. Likewise, it is apparent from Figure 7(b) ((B), (C), and (D)) (treated groups, reverse *β* actin primer) when compared with Figure 7(b) (A) (Control group, reverse *β* actin primer) bases 10 to 20 showed irregular base mismatches. Figure 7(b) (A) (Control group, reverse *β* actin primer) had a poly A tail whereas in Figure 7(b) (B) (7 days' treated group) such tail was tainted in the sequence.

## 4. Discussion

Bioengineered nanoparticles are being considered for a wide range of biomedical applications, from magnetic resonance imaging to drug delivery systems. The development of novel nanomaterials for biomedical applications must be accompanied by careful scrutiny of their biocompatibility. Attention should be paid on the possible interactions between nanoparticles and cells of the immune system. In the present study, DFNPs intended to be used for targeted drug delivery applications were in-house synthesized, characterized, and examined for the immunotoxic potential and cellular response. DFNPs were synthesized using the coprecipitation method. The detailed characterization of DFNPs was carried out using Dynamic Light Scattering for hydrodynamic size profiling, transmission electron microscope for particle size analysis, X-ray diffraction technique for phase purity analysis, thermo gravimetric analysis for quantifying the dextran in DFNPs, and Fourier transform infrared spectral analysis for coating efficiency. The result of the study indicated that the synthesized DFNPs were an authentic particle and have a size less than 25 nm [17, 18].

On entry into the body, nanoparticles can move ahead and get absorbed in liver, spleen, and bone marrow through blood and lymph. It was also known that nanoparticles exposure may result in ROS production and splenocytes apoptosis, change cytokine production, and decrease immune response. Immune cells are the ones that act as defense against any pathogen access. Thus, it is extremely crucial to evaluate the altered immune responses induced upon nanoparticle entry. In this study, systemic immune response induced by DFNPs was evaluated under both* in vitro *and* in vivo *conditions. Spleen is the largest lymphoid organ which plays a critical role in both innate and adaptive immune responses; splenocytes were opted for initial cytotoxicity screening. It was found that no obvious cytotoxicity was noted in spleen cells after DFNPs exposure even though a marginal increase in splenocytes activity was observed. The oxidative stress experienced by the cell was assessed by the measurement of intracellular reactive oxygen species (ROS) level. Increases in intracellular ROS (oxidative stress) are potentially toxic to the cells which, if not neutralized by antioxidant defenses (e.g., glutathione and antioxidant enzymes), could lead to membrane dysfunction, protein degradation and DNA damage, and finally cell death. For measuring the ROS, the cell permeable probe (i.e., 2′,7′-dichlorodihydro-fluorescein diacetate, H_2_DCFDA) was used for the present study. From the results, it was very clear that the ROS generation was well comparable with control values that predict a nontoxic response at the cellular level.

The systemic administration of DFNPs (7, 14, and 21 days) for immunotoxicity evaluation in albino mice showed that the general physical conditions of the experimental animals were normal during the experimental period. All the animals were sacrificed and there were no abnormalities observed during the gross necropsies of animal at the end of each observation period. The results of the proliferation assay indicated that there was a significant reduction in the ^3^H-Thymidine incorporation in both the T and B cells after 14 and 21 days after exposure of DFNPs which needs further investigation.

The concentration of MDA in biological materials has been widely utilized as an indicator of oxidative damage to unsaturated lipid. Measurement of MDA, the byproduct of LPO, provides an exact and well-established index of oxidative damage since it is very reactive and takes part in cross-linking with biomolecules [32]. It is well known that lipid peroxidation occurs naturally in small amounts in the body, mainly by the effect of several ROS or by the action of several phagocytes [33, 34]. In the present study, no changes in the level of lipid peroxidation in liver were observed. This correlates with the results obtained in* in vitro *study using fluorescent probe. But there was a significant reduction of GSH observed after 21 days of exposure and this may be the result of direct reaction of DFNPs with GSH. [35] reported that after administration of nanoparticles; GSH can act as a conjugating agent in their metabolism. When these nanoparticles induce oxidative stress by generating H_2_O_2_ or hydroperoxides, GSH can also be oxidized in a reaction catalyzed by GSH-Px. The depletion of GSH in tissues leads to impairment of the cellular defense against ROS, and may result in peroxidative injury [35, 36].

Regarding the antioxidant enzymes, it was observed that the level of GR and SOD was significantly altered 21 days after exposure period. Intracellular GSH scavenge free radicals formed inside the cells. The cells replenish the lost GSH by converting the peroxides formed from free radicals into GSH. This reaction was catalyzed by GR. Thus, in the present study, the increase in GR activity observed after 7 days of exposure may result in the conversion of peroxides formed by ROS into GSH. After 21 days of exposure, although a slight decrease in GR activity was observed, it was significantly higher compared to control. It was reported that GPx, GR, and SOD protect cells against ROS [37]. GR and GPx are the two most important enzymes in the GSH-GSSG cycle and may be activated by increased hydrogen and/or lipid peroxide production. Antioxidant activity of GSH-Px involves neutralization of H_2_O_2_, reduction of lipid hydroperoxidases, and maintenance of normal membrane permeability [38]. It was also observed that there was no increase in the GPx production, when DFNPs were exposed to mice. The SOD level was also comparable to control values in mice exposed to DFNPs.

The finding of the cytokines analysis of the present study are in line with the literature suggesting that tumor necrosis factor-beta (TNF *β*) is a cytokine that is inhibited by interleukin 10. It was observed that the increase in the concentration of DFNPs increases the expression of IL-10, an anti-inflammatory cytokine, and decreases the expression of TNF *β* which mediates a large variety of inflammatory, immunostimulatory, and antiviral responses [39]. Earlier studies revealed no macrophage or dendritic cell secretion of proinflammatory cytokines when silicone-coated iron oxide nanoparticles were administered [40]. Similarly, the present study revealed that no increase in proinflammatory cytokines occurs upon administration of DFNPs.

Sequence analysis of cytoskeletal *β* actin revealed regions from 20 to 50 bases were conserved in treated mice. Earlier studies of cytoskeletal *β* actin mRNA sequencing of mouse provided information about the conserved sequences as well as the posttranscriptional regions in the sequence [41]. The conserved sequence of *β* actin in mice for miR-644a target site was provided in 2012 [42]. The 5′ end of the mouse *β* actin gene contains sequence elements which mediate the stimulatory effects of serum growth factors and are responsive to both positive and negative regulators of gene expression of several genes [43]. Base mismatches in the nonconserved region observed may not significantly lead to mutations and altered protein synthesis but may result in the short frame shift mutations.

## 5. Conclusion

Animals appeared to be normal during the course of the experimental period after exposure to DFNPs. The DFNPs (<25 nm) are found to be noncytotoxic in spleen cells. It was also evident that the DFNPs do not influence cellular proliferation, LPO, GSH, and antioxidant enzymes. Hence, it can be concluded that the DFNPs were nontoxic at cellular level and nonimmunotoxic when exposed to albino mice, under laboratory conditions simulation. Base alterations in the nonconserved region may not significantly lead to mutations and distorted protein synthesis of the cytoskeletal *β* actin gene but might result in the diminutive frame shift mutations.

## Figures and Tables

**Table 1:** The mouse oligonucleotide forward and reverse primer sequence used to determine specific m-RNA gene expressions.

Number	Primer	Primer sequence	Accession number
1	Interleukin 10 (IL-10)	f-CCAGTCGGCCAGAGCCACAT	NM 010548.2
		r-GGCCATGCTTCTCTGCCTGGG	

2	Interleukin 1 (IL-1)	f-CTCTCCCCAGCTTTTCCAGG	NM 001177975.1
		r-TCTCTGGGCTTGACTGCTTG	

3	Tumour necrosis factor beta (TNF *β*)	f-TGCCAGCTCCAGGATTTCAG	NM 011610.3
		r-CTCAGCCCTCACTTGACCTG	

5	Beta actin (*β* actin)	f-GCGTGGGGACAGCCGCATCTT	BC 023196.1
		r-ATCGGCAGAAGGGGCGGAGA	

## Abbreviations

DFNPs: dextran coated ferrite nanoparticles
ROS: reactive oxygen species
LPO: lipid peroxidation
GSH: reduced glutathione
GR: glutathione reductase
GPx: glutathione peroxidase
SOD: superoxide dismutase
IL1: interleukin 1
IL10: interleukin 10
TNF *β*: tumor necrosis factor *β*.

## References

[1] Corot C., Robert P., Idée J.-M., Port M. (2006). Recent advances in iron oxide nanocrystal technology for medical imaging. *Advanced Drug Delivery Reviews*.

[2] Kumar A., Sahoo B., Montpetit A., Behera S., Lockey R. F., Mohapatra S. S. (2007). Development of hyaluronic acid-Fe_2_O_3_ hybrid magnetic nanoparticles for targeted delivery of peptides. *Nanomedicine: Nanotechnology, Biology, and Medicine*.

[3] Hovgaard L., Brøndsted H. (1996). Current applications of polysaccharides in colon targeting. *Critical Reviews in Therapeutic Drug Carrier Systems*.

[4] Arbab A. S., Bashaw L. A., Miller B. R. (2003). Characterization of biophysical and metabolic properties of cells labeled with superparamagnetic iron oxide nanoparticles and transfection agent for cellular MR imaging. *Radiology*.

[5] Bahadur D., Giri J. (2003). Biomaterials and magnetism. *Sadhana*.

[6] Kim D.-H., Nikles D. E., Johnson D. T., Brazel C. S. (2008). Heat generation of aqueously dispersed CoFe_2_O_4_ nanoparticles as heating agents for magnetically activated drug delivery and hyperthermia. *Journal of Magnetism and Magnetic Materials*.

[7] Gleich B., Weizenecker J. (2005). Tomographic imaging using the nonlinear response of magnetic particles. *Nature*.

[8] Wang Y. X., Hussain S. M., Krestin G. P. (2001). Superparamagnetic iron oxide contrast agents: physicochemical characteristics and applications in MR imaging. *European Radiology*.

[9] Deb S., Giri J., Dasgupta S., Datta D., Bahadur D. (2003). Synthesis and characterization of biocompatible hydroxyapatite coated ferrite. *Bulletin of Materials Science*.

[10] Tan M.-H., Commens C. A., Burnett L., Snitch P. J. (1996). A pilot study on the percutaneous absorption of microfine titanium dioxide from sunscreens. *Australasian Journal of Dermatology*.

[11] Lunov O., Syrovets T., Büchele B. (2010). The effect of carboxydextran-coated superparamagnetic iron oxide nanoparticles on c-Jun N-terminal kinase-mediated apoptosis in human macrophages. *Biomaterials*.

[12] Desai K. G., Park H. J. (2006). Preparation, characterization and protein loading of hexanoyl-modified chitosan nanoparticles. *Drug Delivery*.

[13] Sutton A., Harrison G. E., Carr T. E. F., Barltrop D. (1971). Reduction in the absorption of dietary strontium in children by an alginate derivative. *International Journal of Radiation Biology*.

[14] Elsabahy M., Wooley K. L. (2013). Cytokines as biomarkers of nanoparticle immunotoxicity. *Chemical Society Reviews*.

[15] Gao N., Zhang Q., Mu Q. (2011). Steering carbon nanotubes to scavenger receptor recognition by nanotube surface chemistry modification partially alleviates NF*κ*B activation and reduces its immunotoxicity. *ACS Nano*.

[16] Elsabahy M., Samarajeewa S., Raymond J. E., Clark C., Wooley K. L. (2013). Shell-crosslinked knedel-like nanoparticles induce lower immunotoxicity than their non-crosslinked analogs. *Journal of Materials Chemistry B*.

[17] Mohanan P. V., Geetha C. S., Syama S., Varma H. K. (2014). Interfacing of dextran coated ferrite nanomaterials with cellular system and delayed hypersensitivity on Guinea pigs. *Colloids and Surfaces B: Biointerfaces*.

[18] Mohanan P. V., Syama S., Sabareeswaran A., Sreekanth P. J., Varma H. K. (2014). Molecular toxicity of dextran coated ferrite nanoparticles after dermal exposure to Wistar rats. *Journal of Toxicology and Health*.

[19] Syama S., Reshma S. C., Leji B. (2014). Toxicity evaluation of dextran coated ferrite nanomaterials after acute oral exposure to Wistar rats. *Journal of Allergy & Therapy*.

[20] van de Loosdrecht A. A., Beelen R. H. J., Ossenkoppele G. J., Broekhoven M. G., Langenhuijsen M. M. A. C. (1994). A tetrazolium-based colorimetric MTT assay to quantitate human monocyte mediated cytotoxicity against leukemic cells from cell lines and patients with acute myeloid leukemia. *Journal of Immunological Methods*.

[21] Gayathri V., Geetha C. S., Mohanan P. V. (2013). Protective mechanism of melatonin on kainic acid induced immune modulatory effect on lymphocytes derived from mouse spleen. *Journal of Clinical & Cellular Immunology*.

[22] Gayathri V., Geetha C. S., Mohanan P. V. (2013). Attenuation by melatonin of kainic acid-induced alterations in the expression of mRNAs of some immune-modulatory cytokines, and mitochondrial DNA damage in mice. *The Journal of Toxicology and Health*.

[23] Lowry O. H., Rosebrough N. J., Farr A. L., Randall R. J. (1951). Protein measurement with the Folin phenol reagent. *The Journal of Biological Chemistry*.

[24] Okado-Matsumoto A., Fridovich I. (2001). Subcellular distribution of superoxide dismutases (SOD) in rat liver. Cu, Zn-SOD in mitochondria. *Journal of Biological Chemistry*.

[25] Moron M. S., Depierre J. W., Mannervik B. (1979). Levels of glutathione, glutathione reductase and glutathione S-transferase activities in rat lung and liver. *Biochimica et Biophysica Acta*.

[26] Mize C. E., Langdon R. G. (1962). Hepatic glutathione reductase. I. Purification and general kinetic properties. *The Journal of Biological Chemistry*.

[27] Rotruck J. T., Pope A. L., Ganther H. E., Swanson A. B., Hafeman D. G., Hoekstra W. G. (1973). Selenium: biochemical role as a component of glutathione peroxidase. *Science*.

[28] Marklund S., Marklund G. (1974). Involvement of the superoxide anion radical in the autoxidation of pyrogallol and a convenient assay for superoxide dismutase. *European Journal of Biochemistry*.

[29] Dussault A.-A., Pouliot M. (2006). Rapid and simple comparison of messenger RNA levels using real-time PCR. *Biological Procedures Online*.

[30] Koekemoer T. C., Downing T. G., Oelofsen W. (1998). An alternative PCR assay for quantifying mitochondrial DNA in crude preparations. *Nucleic Acids Research*.

[31] Sanger F., Coulson A. R. (1975). A rapid method for determining sequences in DNA by primed synthesis with DNA polymerase. *Journal of Molecular Biology*.

[32] Kubow S. (1990). Toxicity of dietary lipid peroxidation products. *Trends in Food Science and Technology*.

[33] Hoskins C., Wang L., Cheng W. P., Cuschieri A. (2012). Dilemmas in the reliable estimation of the *in-vitro* cell viability in magnetic nanoparticle engineering: which tests and what protocols?. *Nanoscale Research Letters*.

[34] Cho Y., Shi R., Ben Borgens R. (2010). Chitosan nanoparticle-based neuronal membrane sealing and neuroprotection following acrolein-induced cell injury. *Journal of Biological Engineering*.

[35] Sies H., Akerboom P. M. T. (1984). Glutathione disulfide efflux from cells and tissues. *Methods in Enzymology*.

[36] Berivan T., Nuray N. U. (2006). Kinetic mechanism and molecular properties of glutathione reductase. *FABAD Journal of Pharmaceutical Sciences*.

[37] Flohe L., Pryor W. (1982). Glutathione peroxidase brought to focus. *Free Radicals in Biology and Medicine*.

[38] Zachara B. A., Trafikowska U., Labedzka H., Sosnowski A., Kanarkowski R. (1990). Effect of selenium supplementation on glutathione peroxidase synthesis and element accumulation in sheep erythrocytes. *Biomedica Biochimica Acta*.

[39] Waltenbaugh C., Doan T., Melvold R., Viselli S. (2008). *Immunology*.

[40] Kunzmann A., Andersson B., Vogt C. (2011). Efficient internalization of silica-coated iron oxide nanoparticles of different sizes by primary human macrophages and dendritic cells. *Toxicology and Applied Pharmacology*.

[41] Toicunaga K., Taniguchi H., Yoda K., Shimizu M., Salciyama S. (1986). Nucleotide sequence of a full-length cdna for mouse cytosketetal *β*-actin raRNA. *Nucleic Acids Research*.

[42] Sikand K., Singh J., Ebron J. S., Shukla G. C. (2012). Housekeeping gene selection advisory: glyceraldehyde-3-phosphate dehydrogenase (GAPDH) and *β*-actin are targets of miR-644a. *PLoS ONE*.

[43] Elder P. K., French C. L., Subramaniam M., Schmidt L. J., Getz M. J. (1988). Evidence that the functional *β*-actin gene is single copy in most mice and is associated with 5′ sequences capable of conferring serum- and cycloheximide-dependent regulation. *Molecular and Cellular Biology*.

